# Catch yield and selectivity of a modified scallop dredge to reduce seabed impact

**DOI:** 10.1371/journal.pone.0302225

**Published:** 2024-05-13

**Authors:** Mairi Fenton, Claire L. Szostek, Adam Delargy, Andrew F. Johnson, Michel J. Kaiser, Hilmar Hinz, Natalie Hold, Marija Sciberras

**Affiliations:** 1 The Lyell Centre, Heriot-Watt University, Edinburgh, United Kingdom; 2 Environment and Sustainability Institute, University of Exeter, Penryn Campus, Cornwall, United Kingdom; 3 School of Ocean Sciences, Bangor University, Gwynedd, United Kingdom; 4 School for Marine Science & Technology, University of Massachusetts, New Bedford, Massachusetts, United States of America; 5 Instituto Mediterraneo de Estudios Avanzados, IMEDEA (CSIC-UIB), Esporles, Islas Baleares, Spain; CIFRI: Central Inland Fisheries Research Institute, INDIA

## Abstract

Global scallop fisheries are economically important but are associated with environmental impacts to seabed communities resulting from the direct physical contact of the fishing gear with the seabed. Gear modifications attempting to reduce this contact must be economically feasible such that the catch numbers for the target species is maintained or increased. This study investigated the outcome of reducing seabed contact on retained catch of scallops and bycatch by the addition of skids to the bottom of the collecting bag of scallop dredges. We used a paired control experimental design to investigate the impact of the gear modification in different habitat types. The modified skid dredge generally caught more marketable scallops *per* unit area fished compared with the standard dredge (+5%). However, the skid dredge also retained more bycatch (+11%) and more undersize scallops (+16%). The performance of the two dredges was habitat specific which indicates the importance of adjusting management measures in relation to habitat type. To realize the potential environmental benefits associated with the improvement in catchability of this gear modification, further gear modification is required to reduce the catch of undersize scallops and bycatch. Furthermore we advocate that technical gear innovations in scallop dredging need to be part of a comprehensive and effective fisheries management system.

## Introduction

Wild capture scallop fisheries contributed 811,000 tonnes to global fisheries landings in 2019 [1], of which 26,000 tonnes with a value of £60 million were landed in the UK alone [2]. Many of these fisheries use towed dredges that are designed to have direct physical contact with the seabed and exert multifaceted impacts on marine ecosystems, affecting seabed habitats, scallop populations, and benthic communities [3–5]. Scallop dredging has been reported to significantly alter seabed habitats through physical disturbance of the substrate and a reduction in topographic complexity [4]. The impact on scallop populations and non-target benthic organisms occurs through the removal and/or damage to the biological features and habitats that juvenile scallops and benthic communities rely on and also through the direct removal and mortality of scallops and non-target organisms [4, 6, 7]. Non-target organisms can make up 15–53% of the total catch biomass of a scallop dredge [8]. Returning organisms to the sea after being caught in fishing gear can mitigate some of this impact, but can still result in their death due to physical injuries, stress or increased vulnerability to predators [6, 9, 10]. Stress and physiological effects from being out of the water during sorting on deck can also be fatal [6]. Damage to bycatch species can occur when they encounter scallop dredges on the seabed or inside the dredge bag [6]. These impacts can lead to changes in community structure, affecting individual organisms, populations, and trophic levels [4, 8, 11].

**Fig 1:**
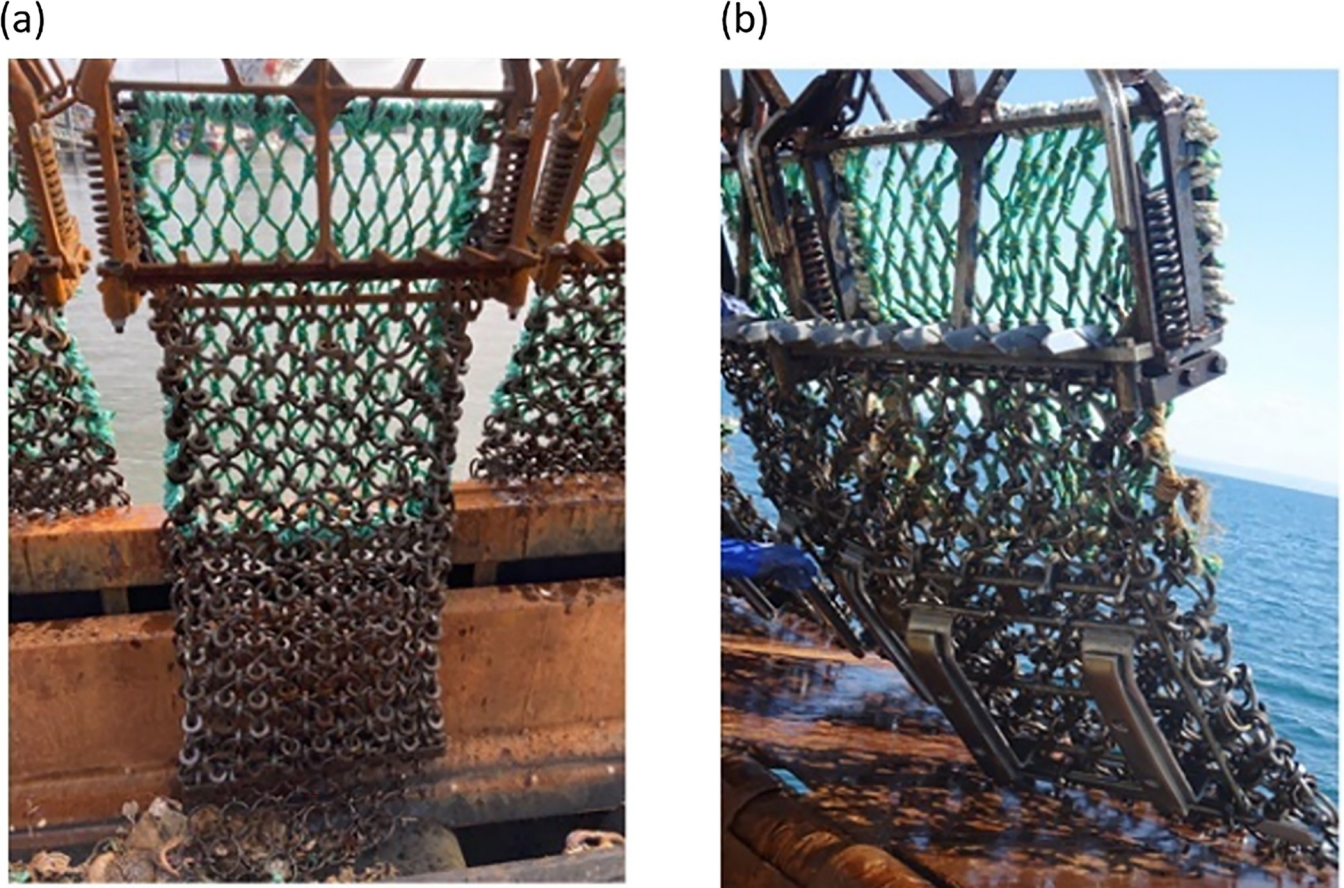
The ‘standard’ Newhaven dredge (a) and modified ‘skid’ dredge (b). The skid frame is attached to the back end of bottom of the collector bag (i.e. furthest away from the mouth of the dredge).

The UK king scallop (*Pecten maximus*) fishery typically uses the spring toothed (or ‘Newhaven’) dredge, the design of which is regulated by the relevant jurisdictions across the country (*e*.*g*. Table 1). A typical ‘gang’ of Newhaven dredges is made up of a heavy steel tow bar to which are attached spring-loaded toothed dredges generally each carrying 8–9 teeth; and a steel collector bag comprising of a belly section made up of interlocking metal rings **(**Fig 1**)**. The dredge teeth have significant impact on the biota as they penetrate the seabed [12], but the contact of the steel collector bags with the seabed is also assumed to have a substantial negative impact on sediment resuspension and benthic fauna [13]. The impact on the seabed and organisms typically increases with the length of the tow as the filling up of catch and stones can substantially increase the weight of the bag (to an estimated 16–78 kg depending on the ground type [14]). Gear modifications that reduce the surface contact between the fishing gear and the seabed could potentially reduce the environmental impacts associated with the use of scallop dredges. Reduction of the physical contact of components of fishing gear has been used in other fisheries such as the introduction of the pulse trawl which replaces tickler chains by lighter electrodes in beam trawl fisheries, the use of semi-pelagic doors in otter trawl fisheries and the addition of cookie cutter disks to sweeps on demersal otter trawls [e.g. 15]. Other modifications in scallop dredge gear such as alteration in overall dredge width and the size of the belly rings and twine top meshes have been reported to increase the yield per recruit for the US sea scallop fishery (*Placopecten magellanicus*), which in turn resulted in a reduction in fishing effort due to the increase in catch rates [3, 16].

**Table 1:** Scallop dredge vessel and scallop dredge characteristics used in the experiment, and regionally relevant legislation.

	MFV Harmoni M147	MFV Evening Star PD1022
Length of vessel (m)	14.9	21.3
Gross registered tonnage	120	160
Engine power (kW)	214	466
Number of dredges *per* side	7	8
Dredge width (cm)	85	76.2
Belly rings (internal diameter, mm)	85	75
Teeth	8 teeth, max length 110 mm	9 teeth, max length 122 mm
Top sheet	Net with mesh size 100 mm	Net with mesh size 100 mm
Relevant legislation	The Scallop Fishing (Wales) (No.2) Order 2010	The Regulation of Scallop Fishing (Scotland) Order 2017

In recent years a number of alternative scallop dredge designs have been tested in the UK, involving modifications either to the dredge teeth (Hydrodredge [17], N-Virodredge [4]) or the collector bag (Oban dredge [18]). These have produced mixed results as to catch numbers and selectivity, reduction in bycatch and gear durability and maintenance. Several other prototypes replacing dredge teeth by rotating cylinders (Magnus effect dredge), or a hydrofoil and water jets (hydraulic dredge) have been described [19]. None of these alternative designs have been used commercially primarily due to low catch efficiency, inconsistency in catch performance, cost of manufacture or practicality [19].

This study investigated the outcome of adding skids to the bottom of the collector bag of scallop dredges on the retained catch of scallops and bycatch in different scallop grounds. The skids were shackled onto the collector bag to give fishers the flexibility of adapting their gear depending on the ground type and lifted the collector bag off the ground by 10 – 11cm (Fig 1). A more detailed description of the gear modification is provided in [20]. It was anticipated that lifting the collector bag off the seabed has the potential to reduce environmental disturbance of scallop dredging by reducing the gear footprint on the seabed and consequently reduce the mortality of benthos in the path of the dredge. Gear modifications attempting to reduce environmental impact must ensure that the practice is both sustainable and economically feasible for the fishers. Failure to do so will reduce the uptake of gear innovations and modifications by the industry. Here, we do not address the environmental disturbance question, but report on the latter question of performance of the modified gear relative to the standard Newhaven dredge with regard to gear catchability and selectivity for marketable and undersized scallops. We also compare catch quality in terms of scallop shell damage and by-catch quantity (biomass of fish and invertebrates other than scallops) and composition as indicators of acute environmental impact of the two fishing gears. The interaction of the fishing gear with the seabed is likely to be influenced by seabed structure and composition, therefore we report on differences in gear catchability and selectivity of the two gears when used in different ground types. The main motivation for the industry to reject new or modified gear stems primarily from the reduction or perceived reduction in target catch, resulting in short-term economic losses [21, 22].

## Methods

### Survey locations

**Fig 2:**
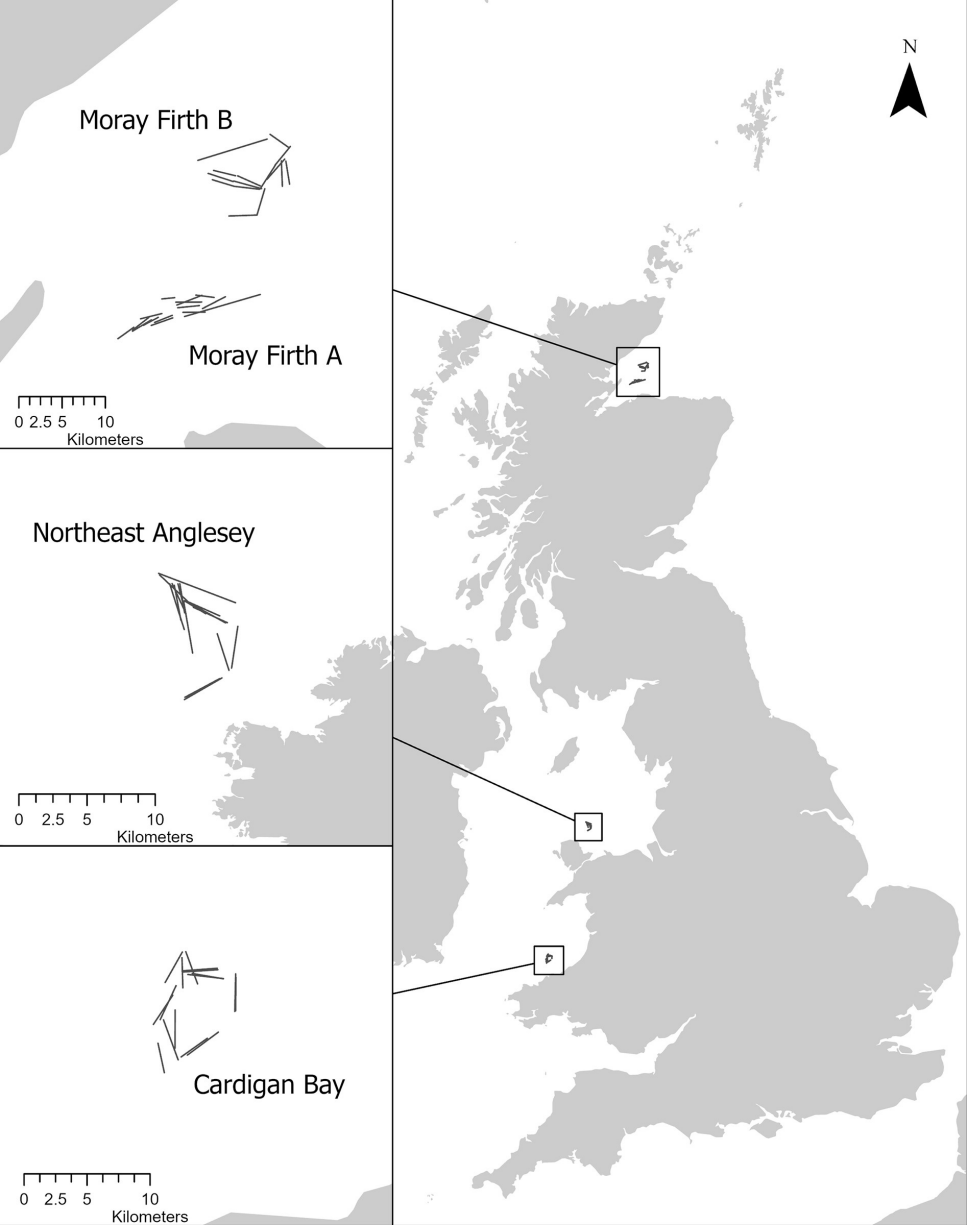
Location of the fishing grounds where fishing trials compared the standard and modified ‘skid’ dredge design. Fishing trials took place in Scottish waters in two areas within the Moray Firth (Moray Firth A: MF_A, Moray Firth B: MF_B) and in two areas in Welsh waters (Northeast Anglesey: W_NEA, Cardigan Bay: W_CB). Sixteen (fifteen in Moray Firth A) replicate hauls were carried out within each area (inset). Basemap created in ArcGIS using freely available data from Natural Earth [23].

Gear trials were carried out in two commercial scallop fishing grounds; one in Welsh waters between 15–21 April 2021 on board the *Motor Fishing Vessel (MFV) Harmoni*, the other in Scottish waters between 22–29 June 2021 on board the *MFV Evening Star* (Table 1). Permission to carry out scientific gear trials using modified scallop dredge gear in Welsh and Scottish waters were obtained from the Welsh Government and from Marine Scotland, respectively, prior to the start of the surveys. At each location fishing was carried out in two areas with different ground hardness. Areas were selected through consultation with skippers to ensure differing ground types and the weight of debris and stones (kg) caught were used as an indicator of the ground type at each survey location (Table 2). In Wales fishing trials were performed outside of the 12 nm limit in Cardigan Bay (W_CB) and along the Northeast coast of Anglesey (W_NEA), in Scotland trials were carried out in two areas in the Moray Firth (MF_A, MF_B) (Fig 2). Trials were carried out in depths ranging from 35-48m.

**Table 2:** Scallop dredge tow characteristics and environmental variables (mean ± SE) at each of the four study areas.

Survey	Area	Number of hauls	Towing speed range (knots)	Sea state (Beaufort scale)	WPUA debris (kg ha^-1^)	WPUA stones (kg ha^-1^)	Tow length (nm)	Depth (m)
**Wales**	W_CB	16	2.8–2.9	**2.1** ± 0.2	**2.8** ± 0.4	**65.4** ± 9.8	**1.5** ± 0.1	**35.9** ± 0.3
	W_NEA	16	2.7–2.8	**1.4** ± 0.1	**2.8** ± 0.2	**107.0** ± 11.9	**1.55** ± 0.1	**46.9** ± 0.7
**Scotland**	MF_A	16	2.6–2.7	**3.7** ± 0.3	**54.4** ± 22.8	**226.2** ± 56.8	**1.42** ± 0.2	**35.9** ± 0.3
	MF_B	15	2.6–2.7	**2.1** ± 0.2	**17.1** ± 3.9	**85.8** ± 42.3	**1.48** ± 0.1	**47.3**± 0.9

### Experimental design

The experiment adopted a paired tow design, whereby the ‘standard’ dredge was towed on one side of the vessel and the modified ‘skid’ dredge towed on the other side of the vessel. This paired gear design was adopted to avoid introducing confounding effects in the data due to variation in sea-state and tidal conditions, towing speed and warp length between different tows and different survey days. To avoid entanglement of the gear when fishing on the seabed, fishers typically paid out more cable (ca. 5 m) on one side than the other. Fishers sometimes report higher catches for the side with more wire. To minimize bias and errors in catch data associated with warp length, the side with more cable paid out was alternated between successive hauls. Fishing was undertaken in line with regional legislation (Table 1).

We performed 16 replicate hauls on each ground type, except for Moray Firth B (MF_B, 15 hauls). Tows were of 30 minutes duration at a mean speed of 2.7 knots (Table 2). Start and end positions of each haul were recorded using vessel GPS at the point when the gear reached the seabed to the point when the winches started to retrieve the gear. The catch was emptied onto the conveyor belts and moved below decks for sorting. The catches from the standard and skid dredges were kept separate and the catch was split into scallops, bycatch species, stones and other debris.

A random subsample of 90 scallops from each gear type and tow were measured for length frequency analysis and scored for shell damage following Jenkins et al. (2001). Shell damage was classified as 4 when the scallop appeared dead or shell crushed, 3 when the shell had large cracks or the hinge was broken, 2 when the edge of the shell appeared chipped, and 1 for no damage. The number and weight of scallops above and below the minimum landing size (> MLS and < MLS, respectively) was recorded for each tow and gear type. MLS for scallops is 110 mm and 105 mm shell height in Welsh inshore (within 12nm) and Scottish waters, respectively. All bycatch was identified to the lowest possible taxonomic level, weighed, and counted by gear type. The weight of stones and other debris (predominantly shell material) was recorded to the nearest gram by gear and habitat type.

### Statistical analyses

All statistical analyses were conducted using ‘R’ [Version 4.1.1, 24]. The data from the two surveys (Scotland and Wales) were analyzed separately due to differences between vessel characteristics, fishing operation and regional legislation (*e*.*g*. different minimum landing sizes).

### Analysis of environmental characteristics of sampling areas

A principal component analysis (PCA) of the four survey areas was undertaken using the ‘prcomp’ function in the ‘stats’ [24] package and the ‘ggbiplot’ [25] package in R, to investigate the difference in environmental characteristics between the areas within the different surveys. The following environmental variables were included: water depth, the weight *per* unit area (WPUA) of debris and stones landed (proxy for ground hardness), sea state and tidal speed.

### Comparison of scallop catch per unit effort

Scallop data were separated into below minimum landing size (MLS; 105mm in Scotland and 110mm in Wales) and marketable scallops (> MLS). The catch data *per* tow were standardized to wet weight *per* unit area (WPUA), using weight (kg) *per* swept area (ha) (where swept areas is the width of the dredge multiplied by the length of the tow). The analysis was also performed for number of scallops caught, but as there was a strong correlation between weight and numbers (S1 Fig), only weight is presented here for brevity. Two out of a total of 63 tows were removed from the analysis as zero scallops were caught in one of either the skid or the standard dredge. The WPUA for the skid dredge was divided by the WPUA of the standard dredge, for each paired tow, to create a relative response ratio, lnRR.


$$lnRR=ln\left(\frac{WPUA\;skid}{WPUA\;standard}\right)$$ (1)

The lnRR quantifies the change in WPUA in the skid dredge relative to the paired standard dredge tow. Positive lnRR values indicate higher WPUA in skid dredges compared to standard dredges, whereas negative lnRR values indicate lower WPUA in skid dredges. lnRR of 0 indicates the same WPUA caught by both skid and standard dredge. The null hypothesis of no difference between dredges can thus be rejected if lnRR is statistically different from zero.

Generalized linear models (GLMs) were used to assess whether environmental or gear parameters influenced the relative WPUA (lnRR) of scallops. Firstly, global models were fitted as Gaussian distributed GLMs (‘stats’ package), which incorporated all the following explanatory variables that could affect the catch:

- *Ground type*. The weight (kg) of debris (WPUA of debris) and stones (WPUA of stones) landed were used as an indicator of the ground type at each tow location. For each tow, we calculated the standardized mean WPUA (kg ha^-1^) (average of weight of debris or stones collected by skid and standard dredges) and used this value in the GLM model to describe ground type for each haul,
- *Sea state* (Beaufort scale). This was included as it can affect fishing efficiency [14] and was determined from wind speed and wave height observations made by the skippers of the vessels for each tow,
- *Tooth length* (cm) of the dredges (Scottish sites only). Tooth length of the dredges can affect the catch efficiency over different ground types (pers. Comm. M Roberts & G Buchan),
- *Tidal speed* at seabed (m/s) was provided by the skipper (Scottish sites only),
- *Warp length* (short or long). This factor was included in the model to determine whether scallop catch by the two gears was influenced by the amount of cable paid out. As noted earlier, warp length was systematically swapped for gear type between successive hauls, to avoid confounding the two effects,
- *Depth*. The water depth (m) in which the fishing operation took place,
- *Area*. To compare the two areas surveyed in Welsh (W_CB vs. W_NEA) and Scottish (MF_A vs. MF_B) waters. Areas were selected in consultation with skippers in order to experience different ground types,
- *Scallop size*. Whether scallops were above (>MLS) or below minimum landing size (<MLS) was included in the model to determine whether there was a difference in impact on undersize scallops between the two dredges.

Tooth length and tidal speed at seabed were not measured during the survey in Wales, hence it was not possible to include these variables in the model for the Welsh data. The interaction between *Area* and *Ground typ*e were included within the model, as well as the interaction between *Tooth length* and *Ground type*.

All combinations of the explanatory variables were tested and compared, with lnRR as the response variable, before being ranked using the Akaike’s information criteria, corrected for small sample sizes (AICc) [26]. The best ranked model, and all models within 2 AICc values, were selected. Using the R packages ‘arm’ [27] and ‘MuMIn’ [28], the parameter estimates and other model values were averaged, creating a final model for each of Scotland and Wales. The suitability of the averaged models was assessed by plotting the model predictions against the observed data and inspected for normality of residuals using the Kolmogorov–Smirnov test and a Q-Q plot. Cook’s distance plots were used to check for outliers. Heteroscedasticity was tested using the Levene’s test and scatter plots of the standardized residuals, fitted values and all covariates were assessed.

### Comparison of scallop catch quality

Physical damage to scallop shells might reduce the commercial value of the marketable scallops and the survivability of undersized scallops. Differences in physical damage endured by scallops from each gear type was examined by calculating the proportion of marketable and undersize scallops within each damage score category for each gear and habitat type. The lowest damage scores (categories 1 and 2) were combined into one category to account for observer variation/categorical bias, as it was often difficult to determine whether damage was score 1 or 2 and there is little difference in physiological damage for the scallop or in marketability between no/slight damage (categories 1 and 2). The remaining categories were considered distinct enough to avoid further statistical issues with ranking bias. Chi-squared analysis was carried out to determine whether the different dredge designs resulted in different damage scores.

### Comparison of scallop size selectivity

To determine if the skid dredge caught significantly more or less scallops of any given length a catch comparison analysis was undertaken using the ‘selfisher’ function in the ‘selfisher’ package in R [29]. The scallop length data were binned into 2mm categories, and this gave sufficient numbers within each size class at the extreme ends of the spectra. The number of scallops of each length class and the proportion of scallops of each length class caught in the skid dredge versus the standard dredge were calculated.

To compare size selectivity, we needed to estimate the skid dredge’s ability to retain scallops of a given size compared to the standard dredge [30]. We modelled the relative retention as a 4^th^-order polynomial, and as a spline with five degrees of freedom using the ‘bs’ function in the ‘splines’ package [24, 29]. Model selection was performed using AIC values to determine the best fit. *Area* was included in the model as an explanatory variable to determine if there were differences in catches among the different ground types. Relative response ratios (Eq (1)) were estimated, with bootstrapped confidence intervals to aid visualization of the results.

### Comparison of bycatch species and stones in dredge catches

The biomass of all bycatch species combined and of different taxonomic classes and species collected from each tow were standardized to WPUA. As for the scallop data, the bycatch WPUA for the skid dredge were divided by the WPUA for the standard dredge, for each paired tow, to give lnRR. Generalized linear models (GLMs) were fit to assess whether environmental parameters influenced the relative WPUA (RR) of bycatch of each taxonomic class. The weight of stones in the skid and standard dredges were standardized to WPUA, and the lnRR was calculated as for the scallop and bycatch data.

## Results

### Survey areas

**Fig 3:**
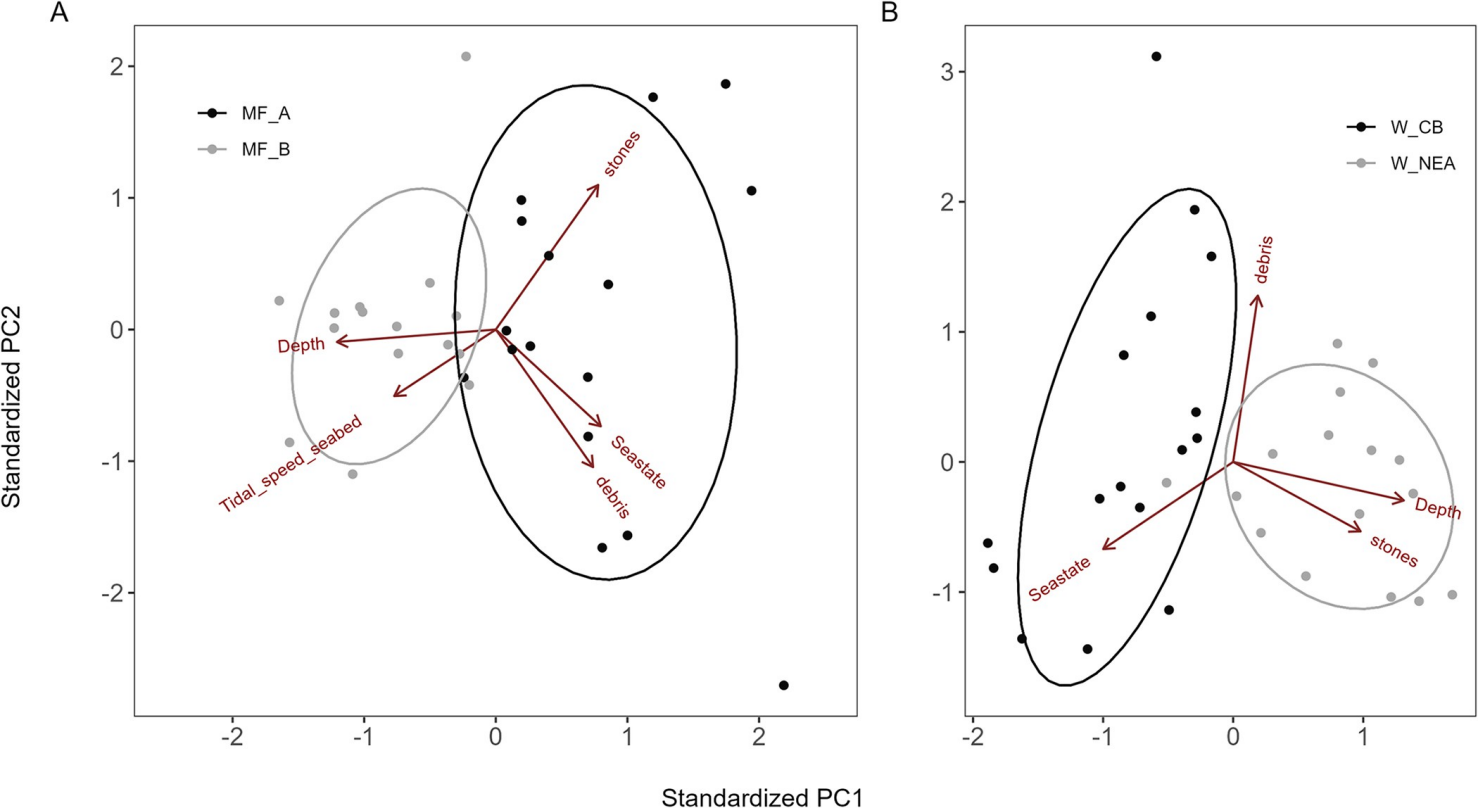
Principle component analysis of the environmental variables experienced during the survey in Scotland (A) and Wales (B) showing the clear differentiation in environmental conditions at each of the two survey sites. The angle between the arrows reflects the correlation between the variables they represent; the smaller the angle the higher the correlation. The first axis (PC1) accounted for 41.0% of the explained variance in the Wales sites (W_CB and W_NEA) and 33.9% in the Scotland survey sites (MF_A and MF_B). The second axis (PC2) accounted for 27.1% in Wales and 27.4% in Scotland. Thus, the first two axes explained 68.1% of the total variance in Wales and 61.3% in the Scotland sites.

A total of 63 tows were conducted across the four areas. The principal component analysis (PCA) examining the environmental characteristics recorded at each site showed clear differences among the two sites surveyed in Wales and in Scotland (Fig 3).

The areas differed primarily in bottom hardness; the fishing grounds surveyed in Northeast of Anglesey (W_NEA) was on average 1.65 times stonier than in Cardigan Bay (W_CB), and site A in the Moray Firth (MF_A) was on average 2.6 times stonier than site B in the Moray Firth (MF_B) (Table 2). Sea state was relatively mild at W_CB, W_NEA, MF_B at the time of survey, but was rougher at MF_A (Table 2).

### Scallop catch yield

**Fig 4:**
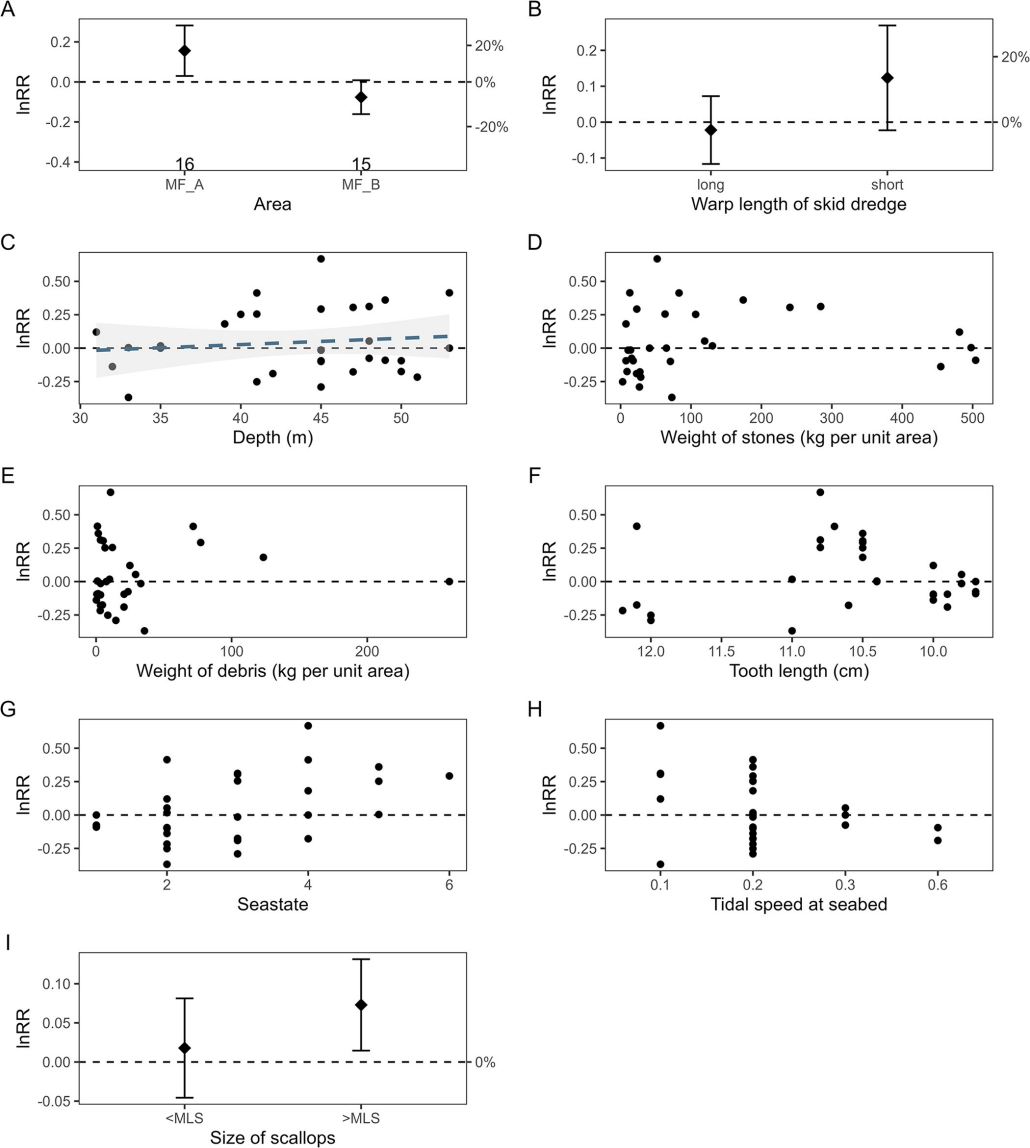
The influence of environment and operation characteristics on the relative catch (response ratio (lnRR) of WPUA, kg ha^-1^ ±95% confidence intervals (CI)) of scallops caught in the Scottish survey areas by skid and standard dredges. The dashed horizontal line (0) represents equal catches between skid and standard dredges. Positive lnRR values indicates higher WPUA in skid dredges compared to standard dredges, negative lnRR values indicates lower WPUA in skid dredges. Error bars in (A) and (B) indicate the 95% CI around the mean lnRR. The number of tows included in the analysis is given below the error bar in the plot (A). Dotted blue lines and grey interval in (C) indicate the fitted relationship and the 95% CI interval for variables that the GLM model analysis found to have a significant influence on the catch ratio between skid and standard dredge (lnRR).

**Fig 5:**
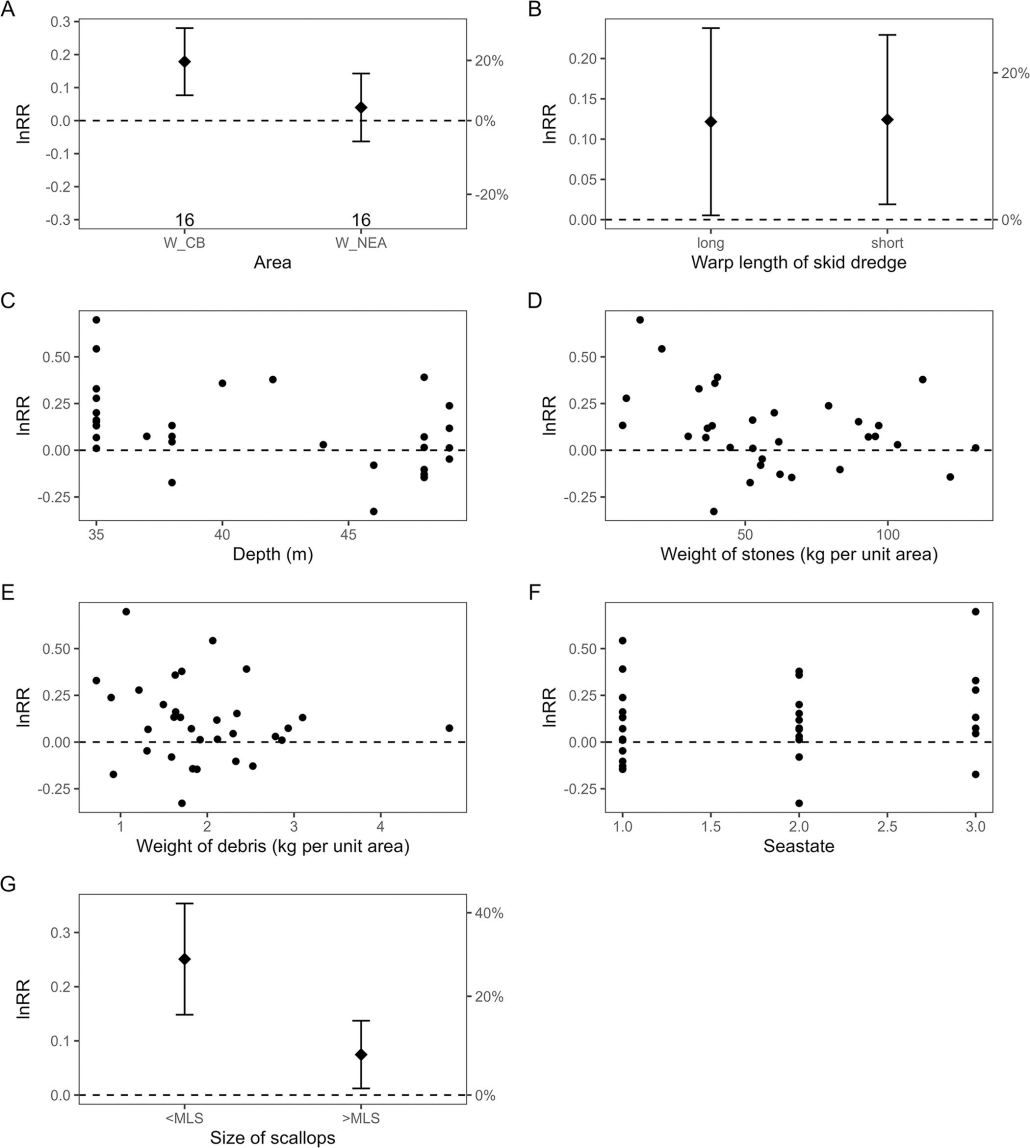
The influence of environment and operation characteristics on the relative catch (response ratio (lnRR) of WPUA, kg ha ^-1^ ±95% confidence intervals (CI)) of scallops caught in the Welsh survey areas by skid and standard dredges.

Site-specific differences were observed in the catch yield of marketable scallops when comparing skid with standard dredges. In Moray Firth A (MF_A) and Cardigan Bay (W_CB) the catch of marketable scallops was significantly higher in skid dredges compared to standard dredges (Figs 4A and 5A). On average, the skid dredges caught 15% more scallops relative to the standard dredge at these two sites, with a minimum and maximum range of 1 to 33% more scallops caught by skid dredges in MF_A and 6 to 25% more scallops in skid dredges in W_CB. Conversely, catch yield did not differ significantly between skid and standard dredge for scallops in site B in the Moray Firth (MF_B) and Northeast Anglesey (W_NEA) and (Figs 4A and 5A). The catch of undersized scallops (< MLS) was generally higher in the skid dredges relative to the standard dredges, significantly so in the Welsh sites (Figs 4I and 5G).

The catch ratio of scallops between skid and standard dredges was not affected by sea state, ground type or warp length in the Welsh survey areas (Table 3, Fig 5B–5G). Significantly higher biomass of scallops waere caught with skid dredges at site A compared to site B in the Moray Firth (Table 3). Site A was characterised by stonier ground and also rougher sea state conditions at the time of the survey (Table 2). There was a significant effect of area and depth on relative WPUA (lnRR) for marketable scallops at the Scottish sites (Table 3, Fig 4B and 4C), but no effect of tooth length, warp length, sea state or tidal speed on catch ratio (Table 3, Fig 4F and 4G).

**Table 3:** The estimated parameters, standard error, T and p values for the best fit generalised linear models describing the relationship between the environmental parameters and the relative catch (lnRR of WPUA, kg/ha) of scallops.

Survey	Parameters	Estimate	Std. Error	T value	P
(a) Wales	(Intercept)	1.45	0.72	2.01	0.05
	Size	-0.18	0.08	-2.14	0.05
	Depth	-0.03	0.02	-1.54	0.13
	Area	0.15	0.23	0.63	0.53
(b) Scotland	(Intercept)	-0.43	0.41	-1.03	0.31
	Size	0.06	0.05	1.13	0.26
	Area	-0.37	0.06	-6.01	<0.001*
	Warp length	0.06	0.05	1.24	0.22
	Depth	0.02	0.01	4.5	<0.001*
	Tooth length	-0.04	0.03	-1.18	0.24
	WPUA of stones	0.001	0.001	0.85	0.4

### Scallop catch quality

Scallops caught with the skid dredges did not experience any increased shell damage. The proportions of marketable and undersize scallops with different damage scores (1&2, 3, 4) did not differ significantly between the skid and the standard dredge (S1 Table).

### Scallop size selectivity

**Fig 6:**
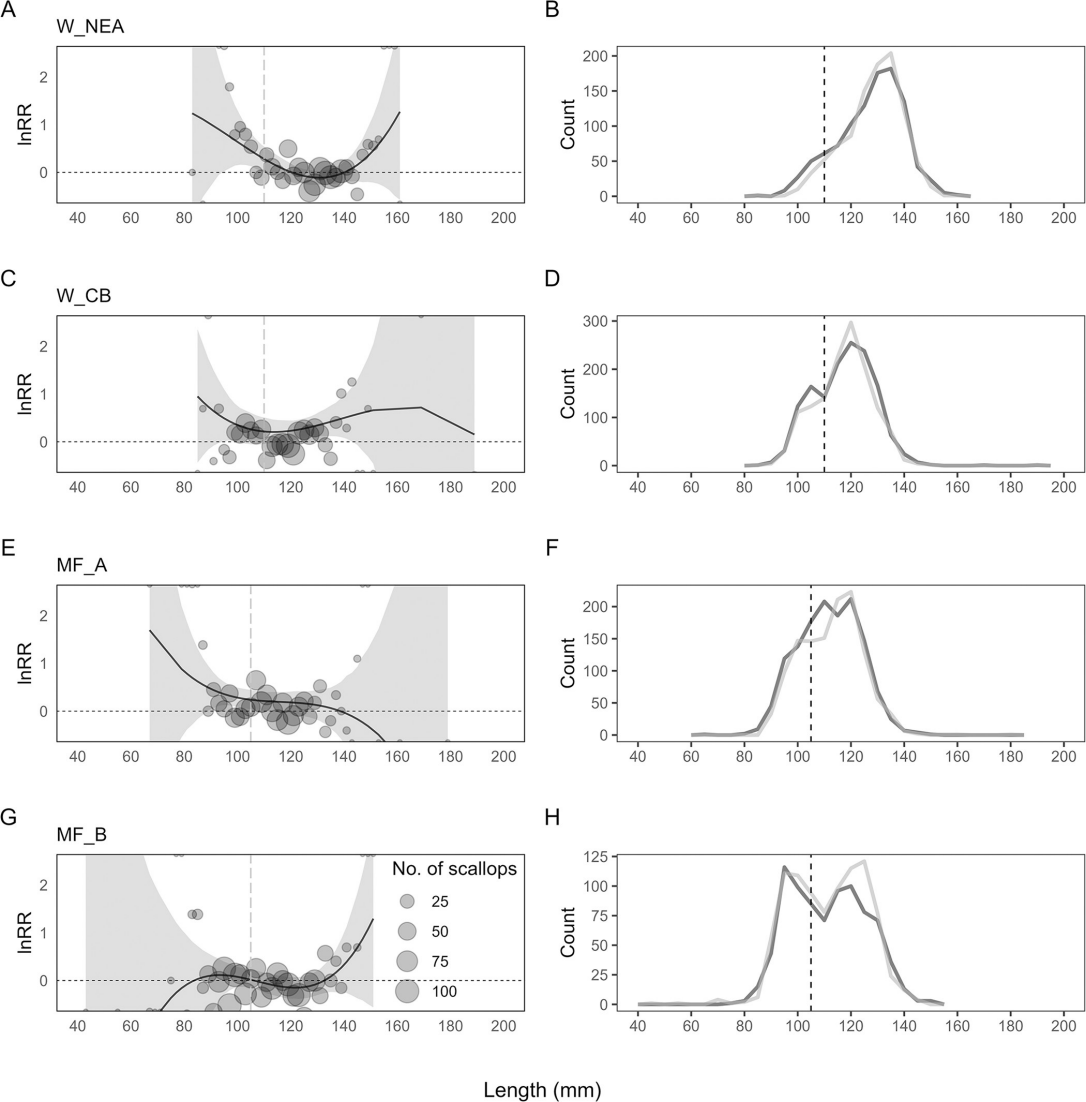
**(A,C,E,G) The modelled ln response ratio, lnRR (± 95% confidence intervals (CI)) curve showing difference between the skid dredge and standard dredge across scallop size classes from the four survey areas.** Bubbles represent number of scallops of each size class. The dotted horizontal line (0) represents equal catches, by abundance, between skid and standard dredges. Significant differences between the catches of the two dredges occur when the confidence interval (grey shading) does not overlap the horizontal dotted line (lnRR = 0). **(B,D,F,H) Size frequency of catch distributions of scallops caught in the skid and standard dredge plotted for each survey area.** The dashed vertical line in all plots represents the minimum landing size in Scotland (105mm) and Wales (110mm).

Catch comparison modelling (Fig 6) indicated that there were differences in scallop size selectivity at the different sites for the skid and standard dredges. In Cardigan Bay (W_CB), the skid dredge caught significantly more marketable scallops in the size range of 120 - 140mm, and in Moray Firth site A (MF_A) it caught more in the range of 105-110mm (Fig 6). However, the skid dredge caught significantly more 95 – 110mm undersized scallops in the Northeast of Anglesey 95–105 mm sized in the Moray Firth Area A (MF_A) (Fig 6; CI does not overlap the lnRR of 0). There was no significant difference in the size of scallops caught between the skid and standard dredge in Moray Firth Area B (MF_B). Direct comparison of number of scallops in different size classes among the four areas are discouraged as the scallop population size composition might be different in different fishing grounds due to varying natural recruitment patterns.

### Bycatch and stones

**Fig 7:**
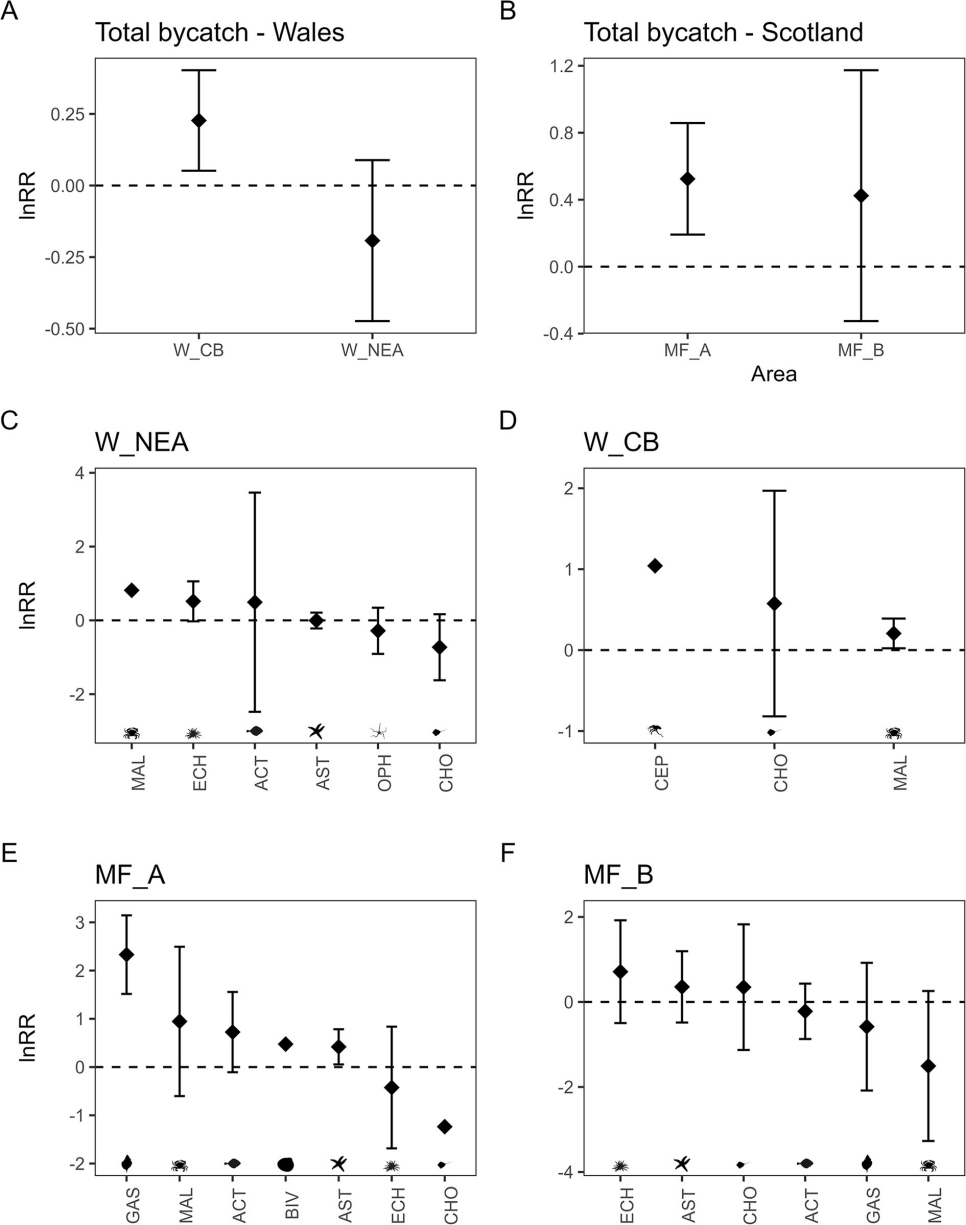
The relative catch (ln response ratio (lnRR) of WPUA, kg ha ^-1^ ±95% confidence intervals (CI)) in skid and standard dredges of all bycatch species combined (A, B) and of different taxonomic groups caught at Welsh (C, D) and Scottish (E, F) sites. ACT = Actinopterygii; AST = Asteroidea; BIV = Bivalvia; CEP = Cephalopoda; CHO = Chondrichthyes; ECH = Echinoidea; GAS = Gastropoda; MAL = Malacostraca; OPH = Ophiuroidea.

The catch composition differed across sites but not between dredges within an area, reflecting different benthic community compositions at the four survey areas (S2 Fig). Skid dredges caught higher bycatch weight relative to the standard dredges at Cardigan Bay (W_CB) and Moray Firth sites A (MF_A) and B (MF_B), however these differences were significant only at W_CB and MF_A (Fig 7A and 7B). On average, bycatch weight was 26% and 70% higher in skid catches in W_CB and MF_A, respectively. The bycatch at Cardigan Bay was primarily made up of the spider crab, *Maja squinado*, which made up more than 90% of total bycatch weight; on average skid dredges caught 23% more spider crabs (Fig 7D). The bycatch at Moray Firth site A (MF_A) was more varied and was primarily composed of asteroids, malacostracans and fish (S2 Fig). Skid dredges caught significantly more fish primarily the European plaice (*Pleuronecta platessa*) and starfish species such as *Crassoster papposus*, *Stichastrella rosea* and *Porania pulvillus* in MF_A (S3 Fig). The standard dredge caught 17% more bycatch weight in Northeast Anglesey (W_NEA), primarily driven by more catches of echinoderms (*Crassoster papposus* and *Ophiothrix fragilis*), however this difference was not significant (Fig 7C, S3 Fig). Interestingly, the standard dredges caught more Chondrichthyes (skates and rays) relative to the skid dredges on stonier grounds at the Scottish study areas (lnRR ± SE = -0.03 ± 0.01, t = -2.811, p = 0.01, S3 Table), and more malacostracans and echinoids than the skid dredges in deeper waters (S3 Table). Two common skates (*Dipturus batis; IUCN critically endangered and Priority Marine Feature in Scotland*) were caught in Moray Firth B (MF_B), both in the skid dredge. In general, the two dredges didn’t differ in their catches of different taxa in Northeast Anglesey (W_NEA) and Moray Firth site B (MF_B) (Fig 7).

**Fig 8:**
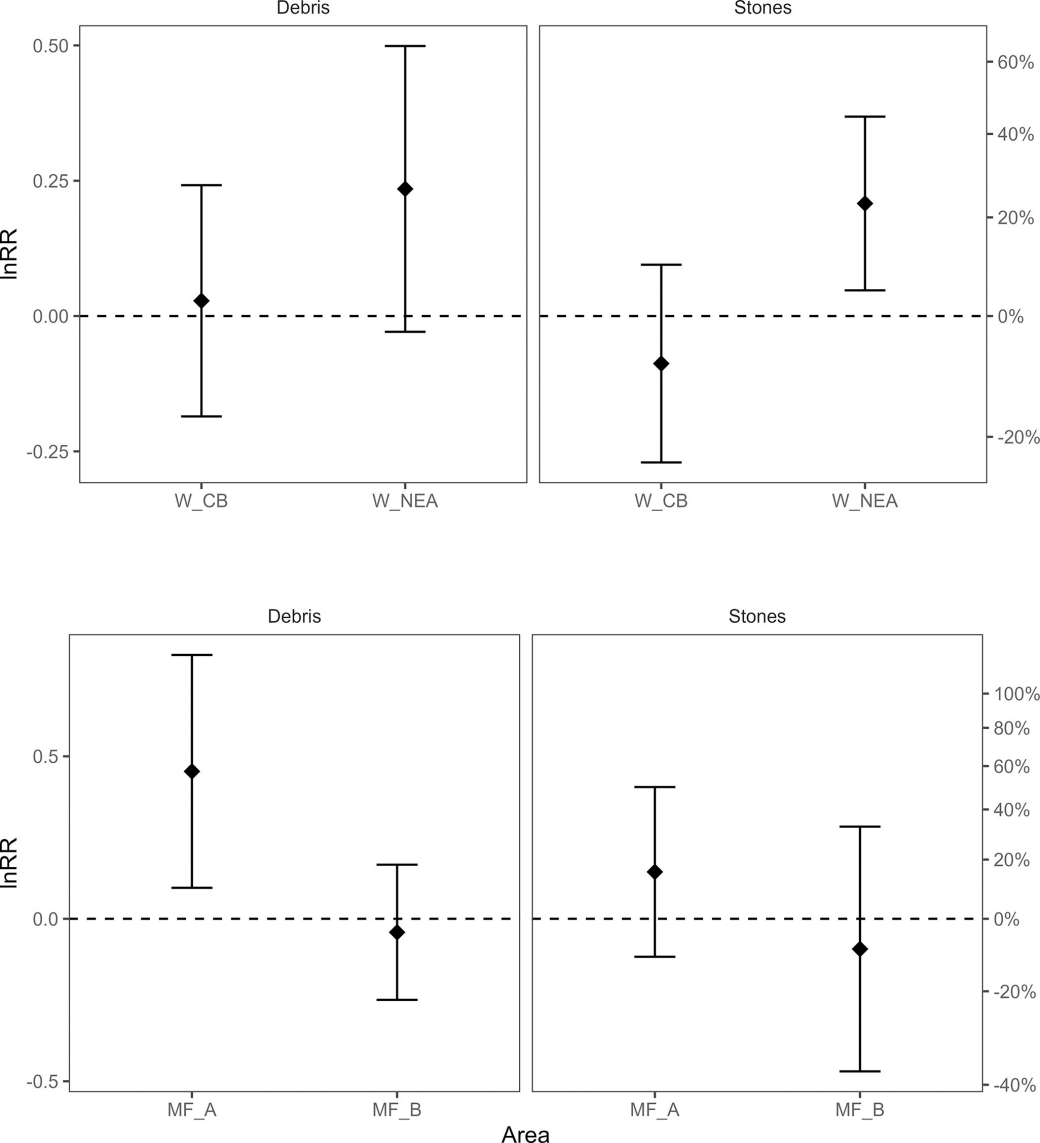
The relative catch (ln response ratio (lnRR) of WPUA, kg ha ^-1^ ±95% confidence intervals (CI)) of debris and stones landed in the skid and standard dredges in each area.

Overall, stone and debris catches did not differ significantly between the two dredge types, except for Northeast Anglesey where the skid dredges caught 23% and 27% more stone and debris, respectively, and at Moray Firth site A where the skid dredges caught 57% more debris (Fig 8). Debris was primarily composed of broken shells.

## Discussion

One of the main barriers to uptake of fishing gear innovation is the potential for loss of catch and income unless this is offset by improved catch quality and/or reductions in overhead costs such as fuel usage [31]. The biomass of marketable scallops caught by the skid dredge was generally higher than the standard dredge. However, the skid dredge also retained more bycatch and undersize scallops. Thus, the benefits of the increased catch rate of marketable scallops could be offset by the negative aspect of retaining additional bycatch and undersized scallops. The issue of increased bycatch could be mitigated to some extent by using a management measure that fixed the upper limit of landed scallops. This would lead to reduced fishing time, reducing the impact on both bycatch and the seabed—the enhanced catch from the skid dredge would help offset the economic consequences of this management approach for fishermen. Higher catch rates of marketable scallops could result in shortened tows which would reduce the impact on the seabed, as this typically increases with the length of the tow [14]. Although catch and bycatch of different tow times / lengths (e.g. 30 mins, 1 hr, 1.5 hrs, 2 hrs) have not been explicitly tested in this study, we would expect there to be a threshold beyond which keeping the gear on the ground for longer does not lead to increased catch. As the bags become fuller with stones, debris and bycatch, gear catch efficiency is expected to drop. From a fisheries management perspective, there is therefore benefit to test how gear catch efficiency varies with tow length for different types of gear. Fishing for longer, does not necessarily result in bigger catches. From an economic (e.g. fuel usage) and environmental (e.g. seabed impact) standpoint, shorter tow times might be best.

The results also demonstrated that the performance of the two dredges was site specific–with differences in the relative catch of scallops (marketable and undersize) and bycatch seen across the study areas. This would suggest that the use of the skid dredges would be most beneficial if deployed in specific geographic areas to maximize catch rates and reduce associated environmental impacts. The reason for higher catches of scallops for the skid dredge (relative to the Newhaven dredge) when fished in deeper waters and heavier sea states is uncertain, but the additional weight of the skids on the dredges is likely to be improving the contact with the seabed and improve catch efficiency in these circumstances. Depth has been associated with reduced gear penetration in the seabed and lower catch efficiency as the pressure exerted on the warps is known to reduce dredge towing traction [5, 32]. Similarly, rough seastate conditions that cause the gear to bounce and lift off the seabed have been associated with lower catch efficiency [15]. The increased weight from the addition of skids to the belly bag may act as a stabilizing force that optimizes interaction of gear with the seabed and therefore result in higher catch efficiency [14]. Spring-toothed scallop dredge catch efficiency is also influenced by substrate type [33–35] with finer substrata generally associated with higher catch rates than coarser substrata [34], although the opposite has been documented in the English Channel [36] where higher catch rates were documented on harder substrate. This study did not find an association between WPUA of stones (an indicator of coarser seabed conditions) and scallop catches for either skid dredges or Newhaven dredges (Table 3). However, the skid dredges did appear to outperform the standard dredges on both clean and stony grounds at opposite ends of a spectrum in terms of ground type, possibly because the increased weight from the skids results in better contact of the dredge teeth with the seabed. One notable difference among these sites was the size of the stones that were retained in the catch. Stones retained at W_NEA were 20–30 cm in diameter, whereas those retained at MF_A were on average 10–15 cm in diameter (personal observation M. Sciberras). This result suggests that there may be a threshold for size of stones beyond which the skid dredge may be no more efficient at catching marketable scallops than the standard gear. The skid dredges did however catch significantly less bycatch than the standard dredge at W_NEA; the elevated belly bag off the ground due to skids together with the increased surface rugosity provided by the bigger stones might thus provide more shelter for some epifauna organisms relative to the standard dredges at this site. Further trials to test the gear performance in difference ground types are recommended.

The increase in catches of undersize (<MLS) scallops reflected the increase in the retained catch of marketable scallops. This increase is concerning as although the direct damage to undersize individuals was observed to be low (1.2% damage score 4 (fatally damaged); 1.2% damage score 3), there is evidence to suggest that cumulative stress events (*e*.*g*. from being repeatedly caught and returned to sea) reduces the reproductive output of individuals as the organism directs more energy towards repair [37]. Maguire, Coleman [38] observed reduced righting and re-burial speed of scallops after dredging which is important as the longer scallops remain un-recessed in sediment the more vulnerable they can be to predation. A reduction in swimming efficiency has also been documented in captured undersize scallops [39] which also increases predation risk. An increase in exposure to air can also result in a negative effect on escape response [39]. Furthermore, the exploitation of a scallop population can change the age structure of the population to domination by younger scallops, resulting in the dependence of the fishery on the strength of the recruiting year-class [40]. Any increase in mortality of young scallops or reduction in reproductive output could therefore have negative implications for the fishery and hence requires appropriate consideration as part of a management plan. Our results suggest that the novel fishing gear modification reported here would benefit the fishery if part of a catch limited management regime.

The composition and proportion of bycatch in scallop fisheries varies considerably at both localised and broad regional scales [8]. Similarly, the mean proportion of bycatch from total biomass caught in this study varied considerably across fishing grounds (Moray Firth A 9.5%; Moray Firth B 14.3%; Cardigan Bay 46%; Northeast Anglesey 23.3%) and generally concurred within proportions recorded in Szostek, Murray [8], suggesting that these patterns of bycatch are consistent features of popular fishing grounds.

The skid dredge caught more bycatch overall (11%), however when looking at differences across taxonomic classes caught, the skid dredge did not consistently catch significantly more, and the results were not consistent across areas (Fig 7). The low overall numbers of individuals of particular species or taxonomic classes may have reduced the statistical power to detect clear differences in bycatch between the skid and standard dredges. The consequences of increased bycatch will vary depending on the survivability of the species, which varies with their physical morphology, longevity and reproductive potential. Echinoids in the catches were often crushed and crabs were missing limbs and experienced cracks in their shells. Kaiser and Spencer [41] found that all crabs caught in trawl nets with cracked carapaces or missing over 50% of their limbs died within 48 hours. A study in Australia found mortality rates of >50% for a spider crab species (*Leptomithrax gaimardii*) when caught as bycatch in a scallop dredge fishery [42]. The longer-term effects of increasing the removal of predators such as starfish (*e*.*g*. in Moray Firth A) or crabs (*e*.*g*. in Cardigan Bay) could lead to trophic impacts and shifts in benthic community composition [8, 43], hence this is an important additional consideration in the context of a management regime.

Although chondrichthyes (skates and rays) and flatfish species did not dominate dredge catches for either dredge type, their catch is still a concern for the fishery, and for the conservation of Endangered, Threatened, Protected (ETP) species. The common skate (*Dipturus batis*) is also considered critically endangered on the IUCN Red List and is a Priority Marine Feature (PMF) in Scotland. Reduction of their capture by the fishery would therefore be desirable. One method that has been demonstrated to reduce capture of fish in a queen scallop trawl fishery is the inclusion of artificial light on trawl nets which significantly reduce bycatch of haddock and flatfish [44]. Similarly, a study focusing on the reduction of flatfish bycatch in an ocean shrimp (*Pandalus jordani*) trawl fishery found that adding LED lights on the trawl fishing line reduced bycatch of sole by up to 69% [45]. The reaction and behavior of species to artificial light is species specific, but the use of LEDs on warp or tow bar could provide a method to reduce the bycatch of skates and rays in scallop dredges.

Artificial lights have been shown to influence scallop behavior as well, with a study on *Placopecten magellanicus* finding that that the addition of artificial lights to a towed survey sled reduced scallop swimming behavior [46]. *Pecten maximus* have been shown to swim towards and into static fishing gear that is illuminated with LED lights [47] (although this is not currently a viable alternative to dredging due to low numbers of scallops caught, it could augment existing static gear crustacean catches). The reaction of organisms to artificial light could be further investigated to reduce bycatch or increase scallop catches.

## Conclusions

A critical requirement of any new gear innovation is that profitability is not impacted or is compensated for in one way or another. From an environmental point-of-view it is important that new gear entering the fishery does not create more damage than the status quo. Whilst the addition of skids to traditional gear belly bags has indicated a benefit to the industry as a result of higher catch of marketable scallops, it comes with the downside of catching more undersized scallops and bycatch species. Whilst we consider skid belly bags to be a step forward in the evolution of lower impact scallop dredges, further modifications such as changes to dredge teeth and belly ring size should be considered and tested. No matter the technical gear innovation, we strongly advocate that unless these are part of an effective fisheries management system, any improvement in catch efficiency or environmental impact from the modified gear will not necessarily lead to a reduction in the overall impact. In the absence of appropriate fisheries management measures that promote sustainable harvest, fishing becomes a frantic overcapitalized race for fish. The sessile nature of scallops makes them more vulnerable to overfishing and can result in significant decreases in catch efficiency and profitability for the industry. Effort and spatial controls and co-management measures (e.g. Territorial User Rights Fisheries—TURFs) are part of a suite of management tools that should be considered. None can be expected to be effective if they are applied in isolation without a comprehensive management framework. For example, the higher catchability of marketable scallops from skid dredges might reap some environmental benefit in terms of reduced seabed disturbance, if this is used under a quota-based system, as a reduced level of effort would be required to catch the quota. In countries where fisheries management for non-quota stocks (such as scallops in the UK) has generally lagged behind that of quota species and the industry’s reputation continues to be affected by environmental impacts and ethical responsibility issues, technical gear modifications are one of several management measures and industry actions that can be taken to reduce impacts on target stocks and the environment, while maintaining an acceptable level of performance for the fishery.

## Supporting information

S1 Fig Correlation between the relative WPUA (lnRR) of scallop biomass and relative NPUA (lnRR) of scallop numbers in all areas. (PDF)

S2 Fig Bycatch composition of the skid and standard dredges in each of the four survey areas. (PDF)

S3 Fig The relative catch (response ratio (lnRR)) of WPUA, kg ha ^-1^ ±95% confidence intervals (CI)) of different bycatch species caught in the skid and standard dredges in each area. The species included in the analysis were the dominant species in each area (calculated as making up the top 80% of total catch by abundance) to reduce sampling bias (missing small/encrusting individuals).
(PDF)

S4 Fig The wear on the collecting bag on ‘standard’ Newhaven (a) and modified ‘skid’ (b) dredges after the Scottish survey.
(PDF)

S1 Table Number of scallops with damage scores 1 & 2, 3 and 4 with chi-squared analysis to examine the impact of dredge type on damage score. (PDF)

S2 Table The estimated parameters, standard error, T and p values for the generalised linear model describing the relationship between the environmental parameters and the relative catch (lnRR of WPUA, kg/ha) of each taxonomic class of bycatch in the Welsh survey. The relationship could not be modelled for every environmental parameter and taxonomic class as relative catch could not be calculated when individuals were not recorded in the same tow in both the skid and standard dredge.
(PDF)

S3 Table The estimated parameters, standard error, T and p values for the generalised linear model describing the relationship between the environmental parameter and the relative catch (lnRR of WPUA, kg/ha) of each taxonomic class of bycatch in the Scottish survey. The relationship could not be modelled for every environmental parameter and taxonomic class as relative catch could not be calculated when individuals were not recorded in the same tow in both the skid and standard dredge.
(PDF)
