# Supplementary material for: Catch yield and selectivity of a modified scallop dredge to reduce seabed impact
Source: PLoS One. 2024 May 13;19(5):e0302225. doi: 10.1371/journal.pone.0302225 (PMC11090360; doi:10.1371/journal.pone.0302225)
Supplement: S1 Fig — (PDF) [file pone.0302225.s001.pdf]

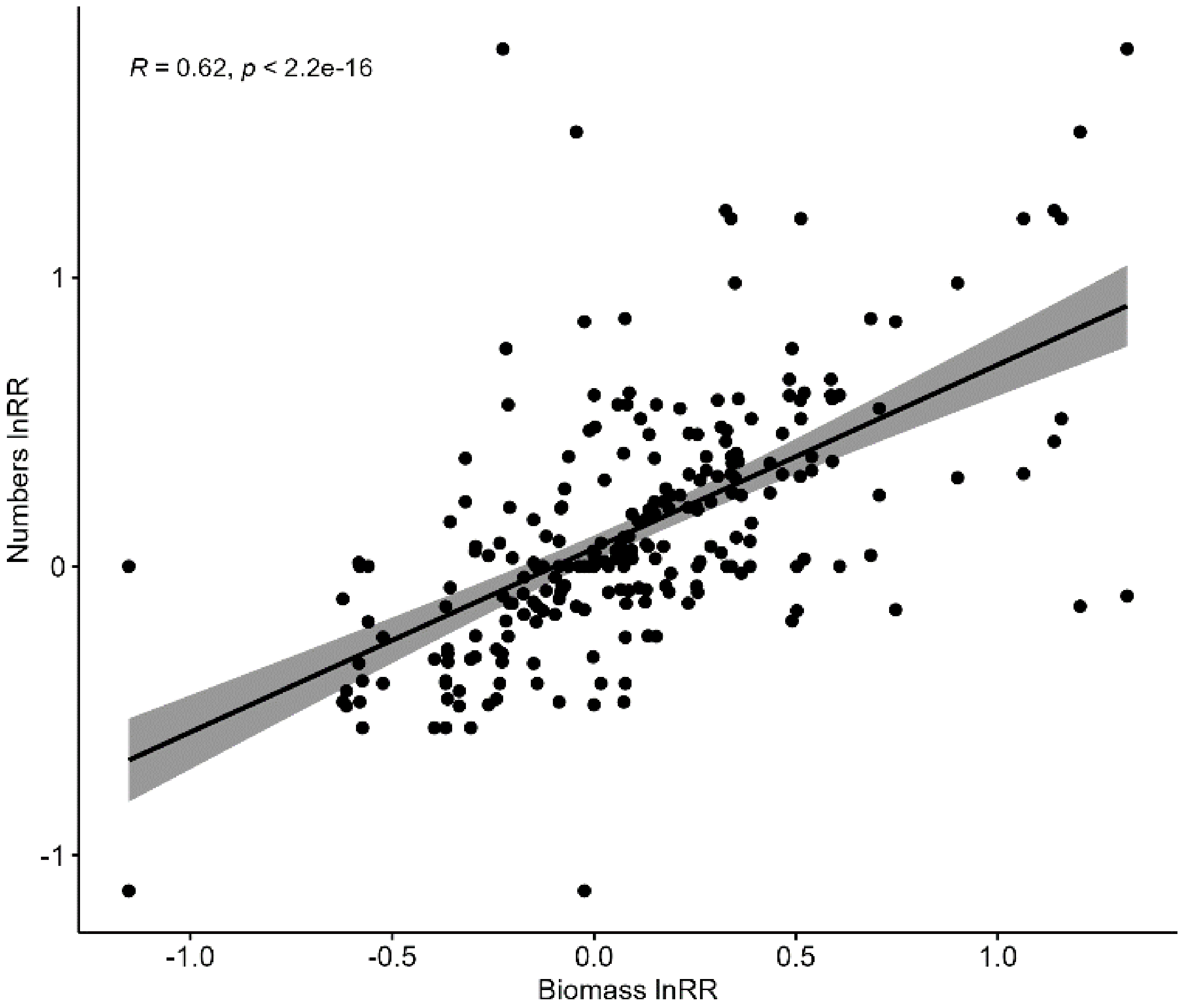

S1 Fig. Correlation between the relative WPUA (lnRR) of scallop biomass and relative NPUA (lnRR) of scallop numbers in all areas.
