# Supplementary figures and images for: Catch yield and selectivity of a modified scallop dredge to reduce seabed impact
Source: PLoS One. 2024 May 13;19(5):e0302225. doi: 10.1371/journal.pone.0302225 (PMC11090360; doi:10.1371/journal.pone.0302225)

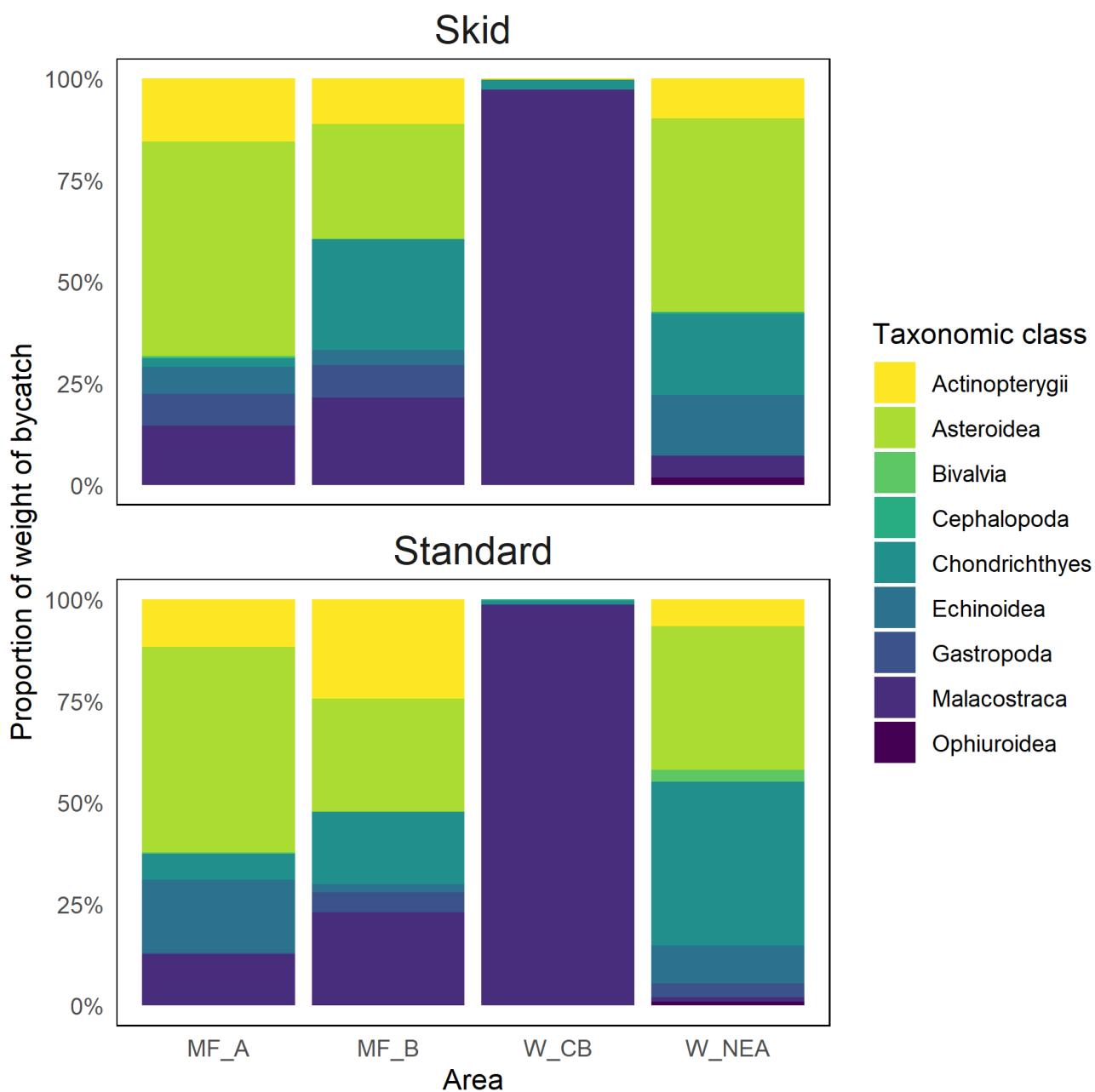

**S2 Fig. Bycatch composition of the skid and standard dredges in each of the four survey areas.**

Supplement: S2 Fig — (PDF) [file pone.0302225.s002.pdf]
