# Supplementary material for: Catch yield and selectivity of a modified scallop dredge to reduce seabed impact
Source: PLoS One. 2024 May 13;19(5):e0302225. doi: 10.1371/journal.pone.0302225 (PMC11090360; doi:10.1371/journal.pone.0302225)
Supplement: S3 Fig — The species included in the analysis were the dominant species in each area (calculated as making up the top 80% of total catch by abundance) to reduce sampling bias (missing small/encrusting individuals). (PDF) [file pone.0302225.s003.pdf]

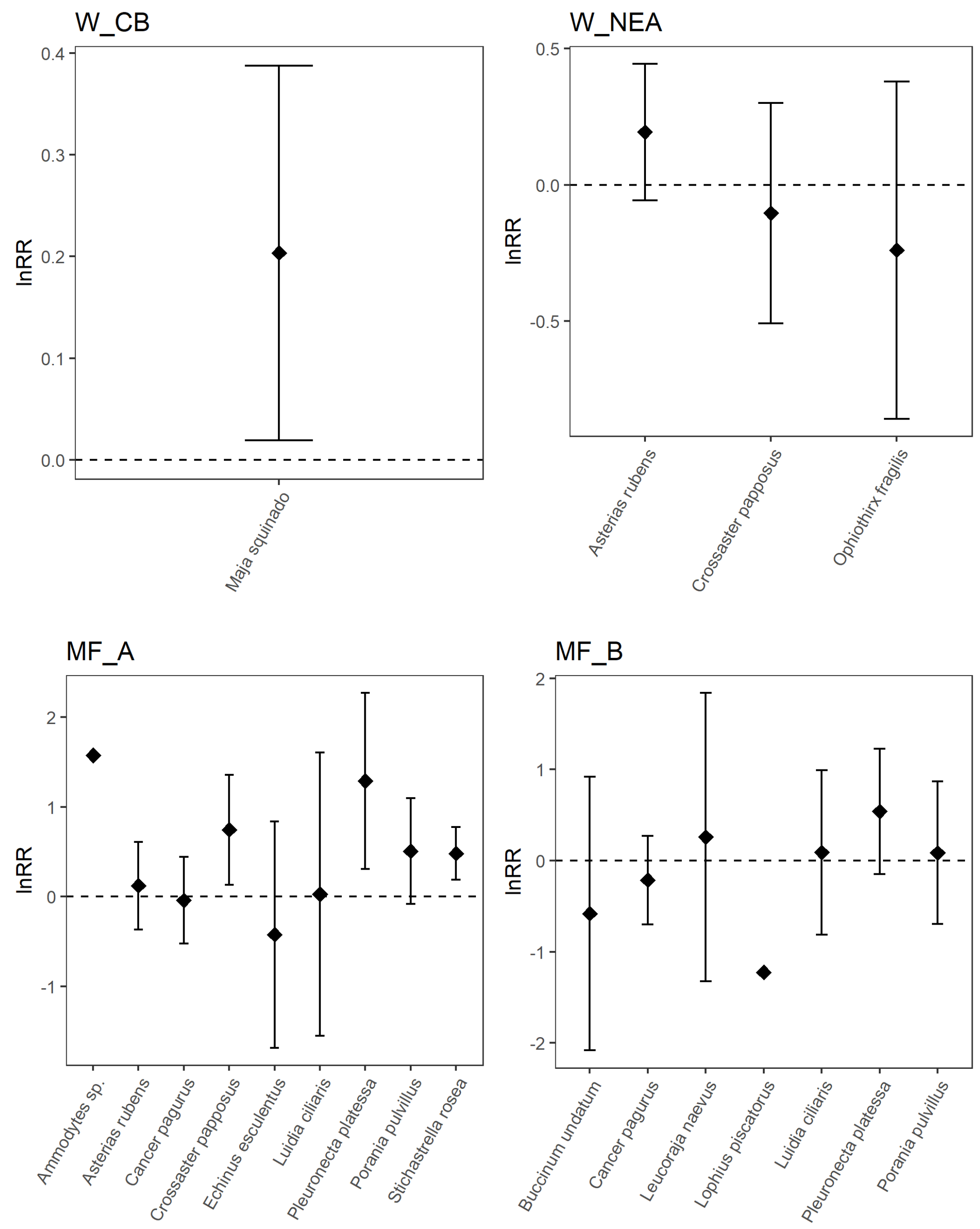

S3 Fig. The relative catch (response ratio (lnRR)) of WPUA, kg ha $^{-1}$ ±95% confidence intervals (CI)) of different bycatch species caught in the skid and standard dredges in each area. The species included in the analysis were the dominant species in each area (calculated as making up the top 80% of total catch by abundance) to reduce sampling bias (missing small/encrusting individuals). The dashed horizontal line (lnRR = 0) represents equal catches by WPUA of bycatch between skid and standard dredges. Positive lnRR values indicates higher WPUA of bycatch in skid dredges compared to standard dredges, negative lnRR values indicates lower WPUA of bycatch in skid dredges. Significant differences occur when the 95% CI does not overlap lnRR = 0.
