# Supplementary material for: Catch yield and selectivity of a modified scallop dredge to reduce seabed impact
Source: PLoS One. 2024 May 13;19(5):e0302225. doi: 10.1371/journal.pone.0302225 (PMC11090360; doi:10.1371/journal.pone.0302225)
Supplement: S4 Fig — The wear on the collecting bag on ‘standard’ Newhaven (a) and modified ‘skid’ (b) dredges after the Scottish survey. (PDF) [file pone.0302225.s004.pdf]

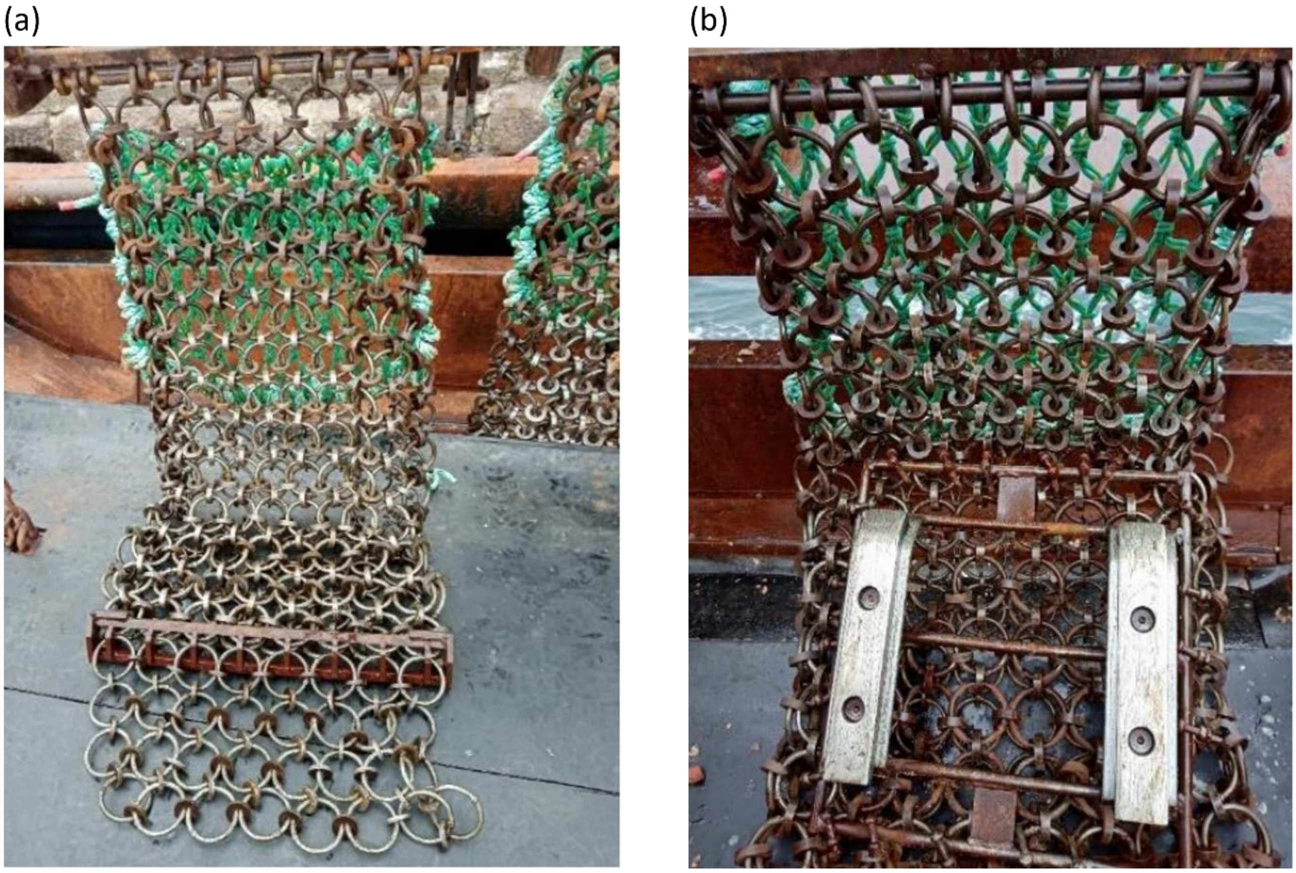

S4 Fig. The wear on the collecting bag on ‘standard’ Newhaven (a) and modified ‘skid’ (b) dredges after the Scottish survey.
