# Supplementary material for: Catch yield and selectivity of a modified scallop dredge to reduce seabed impact
Source: PLoS One. 2024 May 13;19(5):e0302225. doi: 10.1371/journal.pone.0302225 (PMC11090360; doi:10.1371/journal.pone.0302225)
Supplement: S1 Table — (PDF) [file pone.0302225.s005.pdf]

S1 Table. Number of scallops with damage scores 1 & 2, 3 and 4 with chi-squared analysis to examine the impact of dredge type on damage score.

Survey	Area	Size of scallops	Damage score	Number of scallops		Chi-squared	p-value
				Skid	Standard		
(a)Wales	W_CB	>MLS	1 & 2	993	964		
			3	11	22	4.22	0.12
			4	34	38		
		<MLS	1 & 2	387	310		
			3	5	6	1.79	0.41
			4	7	10		
(b)Wales	W_NEA	>MLS	1 & 2	869	880		
			3	5	5	0.04	0.98
			4	31	33		
		<MLS	1 & 2	105	67		
			3	0	0	0.85	0.36
			4	6	1		
(c)Scotland	MF_A	>MLS	1 & 2	873	822		
			3	32	31	0.19	0.91
			4	41	35		
		<MLS	1 & 2	363	324		
			3	12	12	0.09	0.91
			4	4	4		
(d)Scotland	MF_B	>MLS	1 & 2	485	548		
			3	19	13	1.99	0.37
			4	15	13		
		<MLS	1 & 2	301	351		
			3	7	4	1.16	0.45
			4	6	5		
