# Supplementary material for: Catch yield and selectivity of a modified scallop dredge to reduce seabed impact
Source: PLoS One. 2024 May 13;19(5):e0302225. doi: 10.1371/journal.pone.0302225 (PMC11090360; doi:10.1371/journal.pone.0302225)
Supplement: S2 Table — The relationship could not be modelled for every environmental parameter and taxonomic class as relative catch could not be calculated when individuals were not recorded in the same tow in both the skid and standard dredge. (PDF) [file pone.0302225.s006.pdf]

S2 Table. The estimated parameters, standard error, T and p values for the generalised linear model describing the relationship between the environmental parameters and the relative catch (lnRR of WPUA, kg/ha) of each taxonomic class of bycatch in the Welsh survey. The relationship could not be modelled for every environmental parameter and taxonomic class as relative catch could not be calculated when individuals were not recorded in the same tow in both the skid and standard dredge.

	**Estimate**	**Std. Error**	**t value**	**Pr(>|t|)**
Asteroidea	-3.507e+01	1.595e+01	-2.199	0.0355*
Cephalopoda	-3.346e+01	1.413e+01	-2.368	0.0243*
Chondrichthyes	-3.459e+01	1.541e+01	-2.245	0.0321*
Echinoidea	-5.432e+01	1.331e+02	-0.408	0.6860
Malacostraca	-3.448e+01	1.849e+01	-1.865	0.0716.
Ophiuroidea	-3.401e+01	1.811e+01	-1.878	0.0698.
Depth:Actinopterygii	-7.429e-01	3.079e-01	-2.413	0.0219*
Depth:Asteroidea	-1.688e-03	1.575e-01	-0.011	0.9915
Depth:Chondrichthyes	1.361e-02	1.678e-01	0.081	0.9359
Depth:Echinoidea	5.077e-01	2.586e+00	0.196	0.8456
Depth:Malacostraca	9.941e-03	3.475e-01	0.029	0.9774
Depth:Ophiuroidea	4.266e-03	3.064e-01	0.014	0.9890
Debris:Asteroidea	3.087e-01	9.173e-01	0.337	0.7387
Debris:Chondrichthyes	-9.655e-02	8.925e-01	-0.108	0.9145
Debris:Echinoidea	-1.548e+00	3.621e+00	-0.427	0.6720
Debris:Malacostraca	-8.938e-02	3.963e-01	-0.226	0.8230
Debris:Ophiuroidea	-4.797e-01	3.003e+00	-0.160	0.8741
Stones:Asteroidea	1.468e-04	1.402e-02	0.010	0.9917
Stones:Chondrichthyes	-6.542e-03	1.847e-02	-0.354	0.7255
Stones:Echinoidea	-1.531e-02	6.195e-02	-0.247	0.8064
Stones:Malacostraca	-3.692e-03	1.588e-02	-0.233	0.8177
Stones:Ophiuroidea	7.070e-03	3.282e-02	0.215	0.8308
WLshort:Asteroidea:	5.625e-02	8.753e-01	0.064	0.9492
WLshort:Chondrichthyes	8.135e-01	9.670e-01	0.841	0.4066
WLshort:Echinoidea	3.738e+00	2.345e+01	0.159	0.8744
WLshort:Malacostraca	3.554e-01	7.244e-01	0.491	0.6272
WLshort:Ophiuroidea	-6.660e-01	1.424e+00	-0.468	0.6432
Area:Chondrichthyes	-9.037e-01	2.016e+00	-0.448	0.6571
Area:Malacostraca	6.030e-01	4.227e+00	0.143	0.8875
