# Supplementary material for: Catch yield and selectivity of a modified scallop dredge to reduce seabed impact
Source: PLoS One. 2024 May 13;19(5):e0302225. doi: 10.1371/journal.pone.0302225 (PMC11090360; doi:10.1371/journal.pone.0302225)
Supplement: S3 Table — The relationship could not be modelled for every environmental parameter and taxonomic class as relative catch could not be calculated when individuals were not recorded in the same tow in both the skid and standard dredge. (PDF) [file pone.0302225.s007.pdf]

S3 Table. The estimated parameters, standard error, T and p values for the generalised linear model describing the relationship between the environmental parameters and the relative catch (lnRR of WPUA, kg/ha) of each taxonomic class of bycatch in the Scottish survey. The relationship could not be modelled for every environmental parameters and taxonomic class as relative catch could not be calculated when individuals were not recorded in the same tow in both the skid and standard dredge.

	**Estimate**	**Std. Error**	**t value**	**Pr(>|t|)**
Asteroidea	-4.632e-02	9.861e+00	-0.005	0.996293
Bivalvia	-6.672e+00	8.125e+00	-0.821	0.419975
Chondrichthyes	-3.120e+01	2.235e+01	-1.396	0.176065
Echinoidea	8.676e+00	1.121e+01	0.774	0.446709
Gastropoda	-3.596e+01	2.344e+01	-1.534	0.138617
Malacostraca	-1.416e+01	1.613e+01	-0.878	0.389230
Depth:Actinopterygii	-1.791e-03	1.355e-01	-0.013	0.989573
Depth:Asteroidea	-7.286e-03	4.581e-02	-0.159	0.875033
Depth:Chondrichthyes	5.182e-01	4.258e-01	1.217	0.235926
Depth:Echinoidea	-2.360e-01	1.120e-01	-2.108	0.046156*
Depth:Gastropoda	6.243e-01	5.011e-01	1.246	0.225385
Depth:Malacostraca	-5.842e-01	1.827e-01	-3.197	0.004006**
Debris:Actinopterygii	-8.883e-03	7.916e-03	-1.122	0.273399
Debris:Asteroidea	-3.692e-03	4.450e-03	-0.830	0.415222
Debris:Chondrichthyes	5.445e-04	5.612e-02	0.010	0.992343
Debris:Echinoidea	-1.935e-02	2.590e-02	-0.747	0.462623
Debris:Gastropoda	5.834e-02	2.354e-02	2.478	0.020992*
Debris:Malacostraca	-1.255e-02	1.011e-02	-1.241	0.227048
Stones:Actinopterygii	-4.699e-03	6.904e-03	-0.681	0.502962
Stones:Asteroidea	-1.761e-04	1.671e-03	-0.105	0.916972
Stones:Chondrichthyes	-3.376e-02	1.201e-02	-2.811	0.009917**
Stones:Echinoidea	-5.096e-03	4.159e-03	-1.225	0.232878
Stones:Gastropoda	2.021e-03	8.923e-03	0.227	0.822785
Stones:Malacostraca	1.718e-02	9.185e-03	1.870	0.074264.
Seastate:Actinopterygii	5.136e-01	4.220e-01	1.217	0.235936
Seastate:Asteroidea	-1.066e-02	2.208e-01	-0.048	0.961893
Seastate:Chondrichthyes	1.136e+00	9.157e-01	1.240	0.227315
Seastate:Echinoidea	-4.976e-01	5.827e-01	-0.854	0.401933
Seastate:Malacostraca	1.237e+00	8.753e-01	1.413	0.171041
WLshort:Actinopterygii	-8.561e-01	7.195e-01	-1.190	0.246201
WLshort:Asteroidea	-5.643e-01	4.291e-01	-1.315	0.201462
WLshort:Echinoidea	3.494e-01	2.517e+00	0.139	0.890781
WLshort:Malacostraca	-2.833e+00	1.024e+00	-2.767	0.010964*
Toothlength:Actinopterygii	-6.365e-01	3.542e-01	-1.797	0.085477.
Toothlength:Asteroidea	-4.826e-01	4.151e-01	-1.163	0.256888
Toothlength:Echinoidea	-2.953e-01	6.710e-01	-0.440	0.663985
Toothlength:Malacostraca	2.616e+00	6.861e-01	3.812	0.000896***
Tidal_speed_seabed:Actinopterygii	-1.785e+00	2.909e+00	-0.614	0.545465
Tidal_speed_seabed:Asteroidea	-4.603e+00	2.265e+00	-2.032	0.053869.
Tidal_speed_seabed:Malacostraca	7.105e+00	3.734e+00	1.903	0.069634.
Actinopterygii:Area	-6.172e-01	1.402e+00	-0.440	0.663836
Area:Asteroidea	2.852e-01	7.283e-01	0.392	0.698940
Area:Malacostraca	-5.662e-02	2.011e+00	-0.028	0.977781
